# Nanomaterial-Enabled Neural Stimulation

**DOI:** 10.3389/fnins.2016.00069

**Published:** 2016-03-07

**Authors:** Yongchen Wang, Liang Guo

**Affiliations:** ^1^Department of Biomedical Engineering, The Ohio State UniversityColumbus, OH, USA; ^2^Department of Electrical and Computer Engineering, The Ohio State UniversityColumbus, OH, USA; ^3^Department of Neuroscience, The Ohio State UniversityColumbus, OH, USA

**Keywords:** nanotechnology, nanomaterial, neural stimulation, non-contact, noninvasive

## Abstract

Neural stimulation is a critical technique in treating neurological diseases and investigating brain functions. Traditional electrical stimulation uses electrodes to directly create intervening electric fields in the immediate vicinity of neural tissues. Second-generation stimulation techniques directly use light, magnetic fields or ultrasound in a non-contact manner. An emerging generation of non- or minimally invasive neural stimulation techniques is enabled by nanotechnology to achieve a high spatial resolution and cell-type specificity. In these techniques, a nanomaterial converts a remotely transmitted primary stimulus such as a light, magnetic or ultrasonic signal to a localized secondary stimulus such as an electric field or heat to stimulate neurons. The ease of surface modification and bio-conjugation of nanomaterials facilitates cell-type-specific targeting, designated placement and highly localized membrane activation. This review focuses on nanomaterial-enabled neural stimulation techniques primarily involving opto-electric, opto-thermal, magneto-electric, magneto-thermal and acousto-electric transduction mechanisms. Stimulation techniques based on other possible transduction schemes and general consideration for these emerging neurotechnologies are also discussed.

## Introduction

Neural stimulation is an essential technique for restoring lost neural functions and correcting disordered neural circuits in neurological diseases (Hassler et al., 2010). For example, it has exciting applications in the restoration of auditory, visual, bladder and limb functions and the treatment of Parkinson's disease, tremor, dystonia, epilepsy, depression and obsessive-compulsive disorder (Cogan, 2008). Conventional electrode-based, electrical neural stimulation is limited by the strong attenuation of electric fields through tissues and thus often requires surgical placement of the electrodes in an intimate contact to the target neural tissue (Cogan, 2008; Huang et al., 2010). Therefore, it faces challenges such as long-term biocompatibility of the implanted electrodes and surgery-induced trauma (Marin and Fernandez, 2010). Noninvasively applied electrical stimulation, however, suffers from an even poorer spatial resolution, requires a higher power and can cause complications to the intermediate tissues (Histed et al., 2013; Menz et al., 2013).

To address these challenges, noninvasive neural stimulation techniques use light, magnetic fields or ultrasound to directly stimulate neurons in a contactless way (Ueno et al., 1988; Gavrilov et al., 1996; Wells et al., 2005). These techniques have a temporal resolution of milliseconds, but are constrained by a poor spatial resolution (Bolognini and Ro, 2010; Menz et al., 2013). For example, transcranial magnetic stimulation only achieves a spatial resolution at the millimeter scale (Ro et al., 1999; Bolognini and Ro, 2010). The spatial resolution of acoustic neural stimulation highly depends on the ultrasound frequency (Clement et al., 2005; Menz et al., 2013). A relatively high spatial resolution can be achieved for retinal stimulation when a high-frequency ultrasound is used, but stimulation of deep neural tissues such as in the brain requires a low frequency for deep tissue penetration, which leads to a low spatial resolution (Menz et al., 2013).

**Figure 1:**
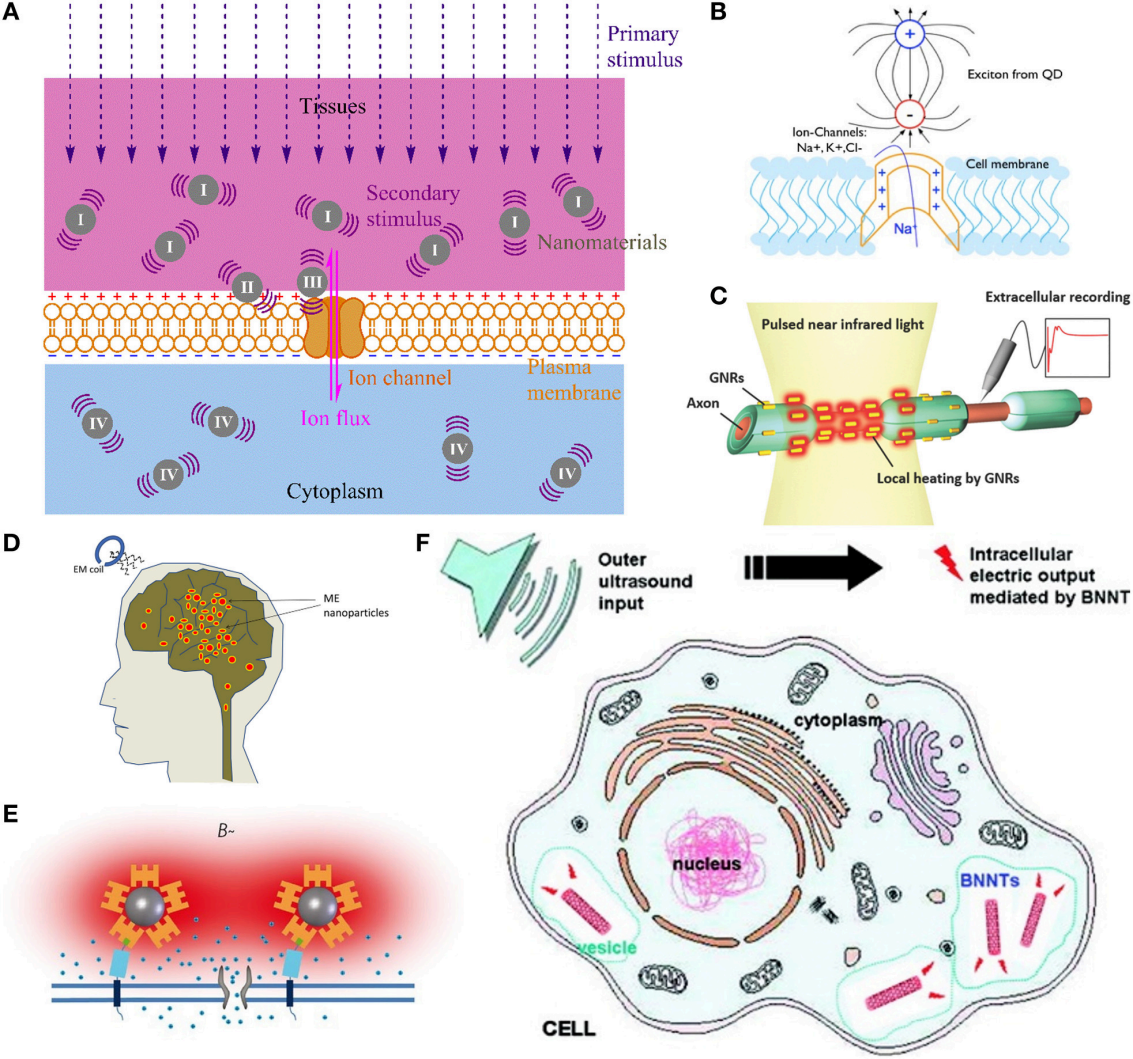
**Principles of nanomaterial-enabled neural stimulation. (A)** General principle: a wirelessly transmitted primary stimulus, such as light, magnetic fields or ultrasound, penetrates through tissues and is converted by the nanomaterial to a localized secondary stimulus, primarily electric fields or heat, at the nanomaterial-neuron interface, to stimulate the neuron. The nanomaterials are (I) dispersed or immobilized in the extracellular environment, (II) attached to the membrane, (III) bound to the ion channel, or (IV) internalized to the cytoplasm. According to the primary and secondary stimuli, nanomaterial-enabled neural stimulation techniques can be classified into **(B)** opto-electric stimulation (Lugo et al., 2012), **(C)** opto-thermal stimulation (Eom et al., 2014), **(D)** magneto-electric stimulation (Yue et al., 2012), **(E)** magneto-thermal stimulation (Huang et al., 2010), and **(F)** acousto-electric stimulation (Ciofani et al., 2010). (Copyright permissions of **B–F** were obtained from the publishers).

Noninvasive or minimally invasive neural stimulation techniques that can be spatially resolved at a near cellular level are greatly desired for clinical diagnosis and treatment of neurological diseases as well as neuroscience studies (Menz et al., 2013). To pursue a minimally invasive neural stimulation technique with a significantly improved spatial resolution, nanomaterials of unique properties are explored as mediators to convert a wirelessly transmitted primary stimulus to a localized secondary stimulus at the nanomaterial-neuron interface, as shown in Figure 1. Additionally, nanomaterials are easy to be surface-modified and bio-conjugated for cell-specific targeting, can be delivered by injection, and can match to the dimensions of subcellular components, such as those of the neuronal membrane and ion channels (Winter et al., 2005; Lugo et al., 2012).

Common primary stimuli also employ light, magnetic fields or ultrasound, which are converted by the nanomaterial to a localized secondary stimulus, primarily electric fields or heat. Localized electric fields stimulate a neuron by perturbing its local transmembrane potential and activating voltage-gated ion channels (Catterall, 1995). Localized heat stimulates a neuron through two proposed mechanisms: the thermal effect on the cell membrane (1) changes the membrane capacitance and/or (2) activates temperature-gated ion channels of the family of transient receptor potential vanilloid (TRPV) channels (Albert et al., 2012; Shapiro et al., 2012; Paviolo et al., 2014b).

This class of nanomaterial-enabled neural stimulation schemes includes, but is not limited to, opto-electric transduction via quantum dots (QDs; Winter et al., 2001, 2005; Gomez et al., 2005; Pappas et al., 2007; Molokanova et al., 2008; Lugo et al., 2012; Bareket et al., 2014), opto-thermal transduction via gold nanomaterials (Paviolo et al., 2013, 2014a, 2015; Eom et al., 2014; Yong et al., 2014; Yoo et al., 2014; Carvalho-de-Souza et al., 2015), magneto-electric transduction via magneto-electric nanoparticles (Yue et al., 2012; Guduru et al., 2015), magneto-thermal transduction via superparamagnetic nanoparticles (Huang et al., 2010; Stanley et al., 2012; Chen et al., 2015), and acousto-electric transduction via piezoelectric nanomaterials (Ciofani et al., 2010; Marino et al., 2015). These schemes are categorized in Table 1 based on their primary stimulus and reviewed in this paper.

**Table 1:** **Transduction schemes of nanomaterial-enabled neural stimulation**.

**Transduction**	**Primary stimulus**	**Secondary stimulus**	**Nanomaterial**	**Placement**
Opto-electric	Light	Electric field	Quantum dots	I or II
Opto-thermal	Light	Heat	Gold nanomaterials	I, II, III, or IV
Magneto-electric	Magnetic field	Electric field	Magneto-electric nanoparticles	Not available
Magneto-thermal	Magnetic field	Heat	Superparamagnetic nanoparticles	I, II, or III
Acousto-electric	Ultrasound	Electric field	Piezoelectric nanomaterials	II or IV

## Nanomaterial-enabled optical stimulation

Optogenetics genetically inserts photosensitive ion channels into a neuron's membrane and modulates the neuronal activity using a blue light (Boyden et al., 2005). This technique has an impressive spatiotemporal resolution and cell-type specificity. However, due to the limited tissue-penetrating capability of the blue light, this method is usually invasive, requiring the implantation of a light source close to the target tissue (Zhang et al., 2010; Jacques, 2013). Noninvasive infrared light is used to directly stimulate neurons without genetic or chemical pre-modification (Wells et al., 2005, 2007). However, the responsivity and sensitivity of this technique need to be further improved (Peterson and Tyler, 2014). Integrating nanomaterials as mediators into optical neural stimulation can help to achieve this goal and also improve the spatial specificity, energy efficiency and safety by using a light source of a significantly lower power (Eom et al., 2014). Opto-electric and opto-thermal stimulations enabled by QDs and gold nanomaterials respectively are two primary types of nanomaterial-enabled optical stimulation techniques and are reviewed below.

### Opto-electric stimulation enabled by QDs

QDs are semiconducting nanoparticles with a diameter from 2 to 6 nm (Algar et al., 2010). Their opto-electric transduction property endowed by quantum confinement makes them suitable as mediators for optical neural stimulation (Winter et al., 2001, 2005; Gomez et al., 2005; Pappas et al., 2007; Molokanova et al., 2008; Lugo et al., 2012; Bareket et al., 2014). Such QD-neuron interfaces have been reviewed in the class of optical neural stimulation techniques elsewhere (Bareket-Keren and Hanein, 2014; Thompson et al., 2014).

At their excitation wavelengths, optically excited QDs generate dipole moments and electric fields (Wang and Herron, 1991; Winter et al., 2001, 2005). Theoretical simulation revealed the possibility of their opto-electric transduction to create adequate localized electric fields to activate voltage-gated ion channels and excite neurons (Figure 1B; Winter et al., 2005; Lugo et al., 2012). Two strategies were used to construct QD-neuron interfaces (Bareket-Keren and Hanein, 2014): the first bound QDs to a neuron's membrane via antibodies or peptides (Placement II in Figure 1A; Gomez et al., 2005; Winter et al., 2005); the second immobilized QDs on a substrate and cultured neurons on top (Placement I; Winter et al., 2005; Pappas et al., 2007; Molokanova et al., 2008; Lugo et al., 2012; Bareket et al., 2014).

An active QD-neuron interface was explored by directly binding antibody- or peptide-conjugated QDs to the neuron's membrane (Winter et al., 2001; Gomez et al., 2005). However, a stable interface for opto-electric transduction was not achieved due to internalization of QDs and nonspecific targeting (Gomez et al., 2005; Bareket-Keren and Hanein, 2014). A subsequent attempt to avoid the internalization problem by tethering QDs to a substrate to create a film only achieved short-term stability (Winter et al., 2005). In a further pursuit of using a QD film to interface with neurons, neuroblastoma NG108 cells were activated to fire action potentials by a photocurrent generated from layer-by-layer assembled, multiplayer films of HgTe QDs (Pappas et al., 2007). In another study, illumination induced membrane depolarization in both nonexcitable and excitable cells and triggered action potentials in NG108 cells and primary hippocampal neurons (Molokanova et al., 2008). Interfaces were also built between a CdTe QD film and prostate cancer LnCap cells, a CdSe QD film and cortical neurons, and a CdSe QD probe and cortical neurons (Lugo et al., 2012). Upon illumination, responding cells were depolarized or hyperpolarized, and action potentials were evoked in the depolarized cortical neurons.

However, the stimulation efficiency and reliability on these QD films still need further improvement (Pappas et al., 2007; Lugo et al., 2012). Even in the best case, only a small portion (e.g., 11%) of the cells was excited (Pappas et al., 2007). Additionally, some neurons were depolarized, whereas others hyperpolarized; and the responses varied considerably among measurements (Lugo et al., 2012). These were improved by composite films through chemically conjugating CdSe/CdS core-shell semiconducting nanorods to carbon nanotubes (Bareket et al., 2014). These films were used to stimulate a chick retina lacking developed photoreceptors under a pulsed light at a wavelength of 405 nm. Their *in vitro* biocompatibility and stability were good for up to 21 days. However, the excitation wavelength was only suitable for superficial stimulation due to limited tissue penetration (Jacques, 2013).

There are also a few other challenges associated with QD-enabled, opto-electric neural stimulation: (1) the strong cytotoxicity of QDs is a concern, particularly when a thin coating is used to achieve an active QD-neuron interface (Derfus et al., 2004; Gomez et al., 2005; Winter et al., 2005; Pappas et al., 2007); (2) the stability of the QD-neuron interface is limited by internalization of QDs via endocytosis (Gomez et al., 2005); and (3) the feasibility of such stimulation schemes needs to be tested *in vivo*.

### Opto-thermal stimulation enabled by gold nanomaterials

In order to generate localized heat to stimulate neurons, microparticles were used as optical absorbers to convert light to heat (Migliori et al., 2012; Farah et al., 2013). The opto-thermal transduction of gold nanomaterials due to localized surface plasmon resonance makes them particularly suitable as optical absorbers for neural stimulation (Figure 1C; Paviolo et al., 2013, 2014a, 2015; Eom et al., 2014; Yong et al., 2014; Yoo et al., 2014; Carvalho-de-Souza et al., 2015). Upon irradiation at the resonant frequency, electrons in gold nanomaterials oscillate and collide, generating and dissipating heat (Roper et al., 2007; Cao et al., 2014). The use of gold nanorods for optical neural stimulation was also reviewed elsewhere (Paviolo et al., 2014b).

Gold nanorods coated with silica were used to stimulate non-genetically modified rat auditory neurons *in vitro* (Placement I and IV; Yong et al., 2014). Illuminated by a pulsed laser at a resonant wavelength of 780 nm, these gold nanorods activated nearby neurons with a linear correlation to the duration of the laser pulse. It was also found that internalized gold nanorods promoted neurite outgrowth and induced a Ca^2+^ influx in NG108-15 cells under continuous and pulsed irradiation respectively, both at a near-infrared resonant wavelength of 780 nm (Placement IV; Paviolo et al., 2013, 2014a, 2015).

*In vivo* optical stimulation of non-genetically modified rat sciatic nerves via gold nanorods was also demonstrated (Placement I; Eom et al., 2014). Illuminated by a pulsed laser at a near-infrared resonant wavelength of 980 nm, sciatic nerves with injected gold nanorods were nearly six times more responsive to fire compound action potentials with a threshold three times lower than the null control. Therefore, the power and exposure duration of the laser stimulus could be greatly reduced, significantly decreasing the risk of tissue damage.

Gold nanoparticles were also used for *in vitro* and *ex vivo* opto-thermal neural stimulation (Carvalho-de-Souza et al., 2015). Gold nanoparticles were conjugated to ligands and specifically targeted to ion channels in the neuron's membrane (Placement III). Upon illumination with light pulses at a visible wavelength of 532 nm, the generated heat depolarized rat dorsal root ganglion neurons and mouse hippocampal slice neurons to fire action potentials. These ion channel-bound gold nanoparticles showed good washout resistance.

For these opto-thermal neural stimulations, internalization of gold nanorods is still a challenge, causing inconsistency, variability and short-term cytotoxicity (Paviolo et al., 2013; Yong et al., 2014). It was reported that an increased pulsed laser irradiance reduced the Ca^2+^ influx induced by internalized gold nanorods (Paviolo et al., 2014a). Inhibitory effects on hippocampal, cortical and olfactory bulb neurons were also observed with gold nanorods electrostatically bound to the neuron's membrane (Placement II; Yoo et al., 2014). Temperature-sensitive inhibitory TREK-1 channels were assumed responsible. Therefore, another challenge is to diverge the different effects in a specific stimulation scheme, so that the neuronal responses can be precisely controlled.

## Nanomaterial-enabled magnetic stimulation

The weak interaction between magnetic fields and tissues enables magnetic fields to penetrate deep into tissues (Huang et al., 2010). However, neural stimulation using magnetic fields usually requires converting the fields to a localized secondary stimulus (Huang et al., 2010). This can be enhanced by magneto-electric nanoparticles via magneto-electric transduction and superparamagnetic nanoparticles via magneto-thermal transduction. These two nanomaterial-enabled magnetic neural stimulation schemes are reviewed below.

### Magneto-electric stimulation enabled by magneto-electric nanoparticles

Magneto-electric nanoparticles, usually made of multiferroics, show a strong magneto-electric coupling and can convert magnetic fields to electric fields due to the magneto-electric effect (Fiebig, 2005). Based on this effect, an idea of using magneto-electric nanoparticles to control voltage-gated ion channels for neural stimulation was proposed (Kargol et al., 2012). Theoretical analysis justified the possibility for deep brain stimulation (Figure 1D; Yue et al., 2012). A proof-of-concept *in vivo* study in mice was conducted using magneto-electric CoFe_2_O_4_-BaTiO_3_ core-shell nanoparticles under a low-intensity magnetic field to modulate deep brain circuits (Guduru et al., 2015). More research is still needed to assess its feasibility with mechanistic specificity and long-term *in vivo* biocompatibility.

### Magneto-thermal stimulation enabled by superparamagnetic nanoparticles

Widely used superparamagnetic nanoparticles can convert alternating magnetic fields to localized heat via magneto-thermal transduction (Laurent et al., 2008), enabling the development of magneto-thermal neural stimulation techniques (Figure 1E; Huang et al., 2010; Stanley et al., 2012; Chen et al., 2015). Streptavidin-conjugated superparamagnetic manganese ferrite (MnFe_2_O_4_) nanoparticles were targeted to the biotinylated peptide of a genetically engineered anchor protein in the membrane of neurons expressing the temperature-gated TRPV1 ion channels (Placement II; Huang et al., 2010). Upon application of a radio-frequency magnetic field, highly localized heating via magneto-thermal transduction induced a Ca^2+^ influx through the TRPV1 ion channels, depolarized the neurons to fire action potentials *in vitro*, and triggered thermal avoidance in worms.

A more specific ion-channel targeting strategy was also implemented in a mouse xenograft model by tethering nanoparticles directly to the TRPV1 ion channels (Placement III; Stanley et al., 2012). 6x-His epitope tag-inserted TRPV1 ion channels were genetically inserted in the cell membrane, and 6x-His epitope tag antibody-conjugated iron oxide nanoparticles were specifically targeted to these ion channels and heated under a radio-frequency magnetic field. The localized heat activated the TRPV1 ion channels and induced a Ca^2+^ influx into the cells faster than in the above work (Huang et al., 2010).

To improve the temporal resolution for neuronal activation and realize a long-term *in vivo* stimulation feasibility, untargeted superparamagnetic iron oxide nanoparticles were dispersed in the vicinity of TRPV1-expressing human embryonic kidney HEK-293FT cells, dissociated hippocampal neurons and neurons at the ventral tegmental area of mice (Placement I; Chen et al., 2015). Upon applying an alternating magnetic field, the magneto-thermally generated heat induced a Ca^2+^ influx in the HEK-293FT cells, stimulated hippocampal neurons to fire action potentials, and activated the neurons at the ventral tegmental area of mice to have an enhanced expression of c-fos, achieving a stimulation response with a latency of 5 s after the onset of the magnetic field. And the stimulation in the mouse model remained effective for at least 1 month thanks to the good biocompatibility, stability and decreased endocytosis of extracellularly dispersed nanoparticles.

Magneto-thermal neural stimulation enabled by superparamagnetic nanoparticles can achieve a uniform stimulation of the target cell population due to the uniform expression of TRPV1 ion channels in these cells across the tissue (Huang et al., 2010; Chen et al., 2015). Although both opto-thermal and magneto-thermal stimulations use heat as the localized secondary stimulus, only the latter has employed genetic modifications to the target neurons for specific targeting and TRPV1 ion channel expression (Huang et al., 2010; Stanley et al., 2012; Chen et al., 2015). The safety of TRPV1 ion channels is, however, concerned, due to their high Ca^2+^ permeability, and thus temperature-gated Na^+^ ion channels are desired as the target (Knöpfel and Akemann, 2010).

## Nanomaterial-enabled acoustic stimulation

As a wirelessly transmitted primary stimulus, ultrasound interacts with tissues weakly and can penetrate deep into soft tissues with minimal energy absorption (Tyler, 2011). It can also be focused at a submillimeter resolution (Gavrilov et al., 1996; Menz et al., 2013; Ibsen et al., 2015). Ultrasound has been directly applied to stimulate both the peripheral and central neural systems, but these techniques are limited by a low energy efficiency and mechanistic non-specificity (Gavrilov et al., 1996; Tyler, 2011; Legon et al., 2014).

### Acousto-electric stimulation enabled by piezoelectric nanomaterials

Piezoelectric nanomaterials can convert ultrasound waves to electric fields via acousto-electric transduction due to their piezoelectricity (Wang and Song, 2006; Wang et al., 2007). Such an acousto-electric transduction may facilitate neural stimulation by a low-intensity ultrasound (Figure 1F; Ciofani et al., 2010; Marino et al., 2015). Neurite outgrowth of PC12 and SH-SY5Y cells was promoted by internalized piezoelectric boron nitride nanotubes under ultrasound stimulation (Placement IV), implying a stimulating effect of the acousto-electric transduction (Ciofani et al., 2010). Piezoelectric barium titanate nanoparticles were electrostatically attached to SH-SY5Y cells and induced Ca^2+^ and Na^+^ influxes in an ultrasonic field (Placement II; Marino et al., 2015). The possibility of neural stimulation via the acousto-electric transduction of piezoelectric nanoparticles was also theoretically justified (Marino et al., 2015). These works showed the promise in using piezoelectric nanomaterials to facilitate noninvasive acoustic neural stimulation. However, direct evidence for neuronal activation has not been established yet. More work is needed to establish the feasibility of this stimulation technique, particularly with primary neurons and in animal models.

## Future directions

Nanomaterial-enabled neural stimulation is an emerging class of neurotechnologies, with numerous exciting breakthroughs in the past decade. As a powerful enabling tool, nanomaterials can be either applied alone or combined with other approaches including synthetic biology to facilitate innovative neural stimulation schemes. These new techniques not only allow non- or minimally invasive neural stimulation of a high spatial resolution and cell specificity, but also improve the safety by significantly reducing the required power of the primary stimulus (Huang et al., 2010; Eom et al., 2014).

Nanomaterials of other transduction mechanisms, such as magneto-mechanical, acousto-mechanical, and opto-optical transductions, are also worth considering for potential development of additional neural stimulation schemes. Magneto-mechanical transduction via magnetic nanoparticles can convert magnetic fields to localized mechanical forces to activate mechanosensitive ion channels such as the TREK-1 channels (Hughes et al., 2005, 2008; Dobson, 2008). Nanomaterial-enabled, acousto-mechanical transduction may be combined with the recently developed sonogenetics (Ibsen et al., 2015) to improve the activation efficiency of genetically inserted membrane mechanosensitive ion channels. Opto-optical transduction via upconversion luminescent nanoparticles, which convert a long-wavelength light to one of a shorter wavelength, may provide a noninvasive alternative to the implanted laser in optogenetics by converting deep penetrating near-infrared light to localized visible light for activating photosensitive ion channels (Jacques, 2013; Berry et al., 2015).

To select the primary and secondary stimuli, several factors are considered. The primary stimulus needs to penetrate tissues deeply, be easy to focus at an appropriate spatial resolution and be safe for long-term exposure. The secondary stimulus needs to be selected according to an adequate expression of the target ion channels in the neuron's membrane. For example, it is not necessary to genetically modify the target neurons with voltage-gated ion channels to use electric fields as the secondary stimulus, whereas, to use heat, the TRPV1 ion channels may need to be genetically inserted into the membrane of target neurons (Huang et al., 2010; Stanley et al., 2012; Chen et al., 2015). Additionally, placement of the nanomaterials (see Figure 1A), which is crucial to the stability of the nanomaterial-neuron interface (Winter et al., 2001, 2005; Gomez et al., 2005; Bareket-Keren and Hanein, 2014; Chen et al., 2015), should be considered in conjunction with possible pre-modification to the target neurons (Huang et al., 2010; Stanley et al., 2012).

This diverse class of nanomaterial-enabled neurotechnologies is still in their early stages of development, with many having only been validated *in vitro*. To move forward, many issues including biocompatibility, stability, consistency, efficiency and reliability will need to be addressed (Gomez et al., 2005; Pappas et al., 2007; Yong et al., 2014; Chen et al., 2015). For a significant period of time, these neurotechnologies will be used primarily as scientific tools for *in vitro* and/or *in vivo* studies. Clinical application is promising, but remains very challenging due to concerns on the safety of nanomaterials, viral vectors for gene delivery, and genetic modification to the target neurons (Manilla et al., 2005; Maynard et al., 2006).

## Author contributions

YW and LG analyzed the relevant published work, designed the perspective and structure, and wrote the manuscript.

### Conflict of interest statement

The authors declare that the research was conducted in the absence of any commercial or financial relationships that could be construed as a potential conflict of interest.
